# In Response

**Published:** 1892-02

**Authors:** 


### IN RESPONSE.

CONTROVERSIES, when carried on for the development of a principle, may be of great value; indeed it may safely be affirmed that no progress can be made without them, but the critical spirit may not always be used with judgment, and our contemporary, the *Dental Cosmos*, will, we hope, pardon us for the opinion that the editorial in the January number, in answer to one of ours in December, partakes largely of that character.

The editorial in the INTERNATIONAL DENTAL JOURNAL was in no sense an effort to lower the reputation of the *Dental Cosmos*, nor was it intended, on the other hand, to elevate the INTERNATIONAL DENTAL JOURNAL.

The text for the article was found in the proceedings of the New York Odontological Society, and was an effort to prove that all journals of a high character "will be read equally and their articles will be copied in proportion to their intrinsic value." That the editor of the *Dental Cosmos* should feel it his duty to controvert this idea seems strange, and yet the spirit and the letter of the editorial indicates an antagonism to the idea of placing the *Dental Cosmos* on an equality with other publications in the interest of dentistry.

We regret that the editor of the *Dental Cosmos*, in the haste to reply to our article, allowed the subjective to overwhelm the objective in his mind, and thereby mistake what he thought we *ought to say*, and place ideas to our credit which do not belong to us, and which a true rendering of the text will not warrant. We quote from the INTERNATIONAL DENTAL JOURNAL of December:

"The question to be considered is, Have we in dentistry a large body of readers? Dentistry is physically a very trying profession, perhaps more so than any other. Very few persons after standing all day over the chair, subjected to the nervous anxieties of patients, are equal to spending their evenings in reading, much less in study. Besides this, it is well known that full practice means constant extra hours of labor, oftentimes far into the night, to keep up with the increasing demands of the clientele. This overtaxing work inevitably decreases the number of readers, and has also had the effect of *limiting* the number of those willing to write for societies and journals."

The reply to this was as follows (*Dental Cosmos* of January):

"Just why the editor of the INTERNATIONAL believes it to be an indisputable fact that there is a *decrease* in the *number of readers* or in the *number of those willing to write for societies* and journals, it is difficult to understand. Our observation and experience point to the opposite conclusion." (Italics ours.)

We are willing to leave these two quotations to the intelligent reader, for it must be quite evident that the idea of a general decrease in readers or writers was not in the mind of the editor of the INTERNATIONAL DENTAL JOURNAL. It is beyond controversy that the conditions named have a depressing effect and has "limited" the number of those "willing to write for societies and journals." We are prepared to recognize the fact that the number of writers at the present time is far in advance of thirty years ago in "quantity" and "quality," but this admission does not controvert the statement made.

The point we desired to impress upon our readers was, that of the six thousand which we arbitrarily estimated as subscribers of journals, not more than half were readers of the scientific matter contained therein, and that this might safely be reduced still further until all the leading journals were placed, in point of value as disseminators of knowledge, upon one common basis. Our critic furnishes no argument to disprove this, but asserts that these journals "are still far from equality when their value as media for the wide dissemination of thought is compared." Exactly how thought is to be disseminated without readers is not stated. Thought goes with culture and understanding, and if the masses in dentistry fail to aspire to a position above mere routine work, how can the larger number of printed pages of the *Dental Cosmos* accomplish it?

There is a broader question underlying this that our critic fails to grasp. It lies in the fact that journals, no matter how large the circulation, if not imbued with the spirit of the specialty they claim to represent, can never make a legitimate impression. Individuals, communities, associations, and occupations are environed by a sphere of thought which, in a measure, antagonizes every other sphere of activity. Neither can enter into that of the other with any hope of successfully arriving at a correct understanding of the needs and relations incident to each. The publisher of books cannot comprehend the author, or the author the publisher. The jeweller regards with a species of contempt the rougher mechanism of the blacksmith, yet each, in their way, are equally important and potent factors in the progress of the age.

It can be said, therefore, without any disparagement, that the commercial journal cannot enter into the spirit or sphere of that one based solely on professional advancement. There is a natural antagonism which, while it is not derogatory to either, remains as a perpetual barrier to assimilation.

We have no conflict with the class of journals the *Dental Cosmos* represents; indeed, we go further and say that dentistry owes, in this country, if not in the world, an immense debt to the publishers of the *Dental Cosmos* and its predecessor, the *Dental News Letter*, for the aid given the dental profession in its days of struggle to something higher. This debt may never be paid properly, but it should always be considered when this subject is presented for discussion. While this is true, and gratefully acknowledged, it does not lessen the duty of dentists to grasp the important fact that this condition of affairs cannot, should not, be eternal. Just so long as dentistry is dependent on commercial journals for the presentation of its ideas, just so long will the spirit of trade, good in its place, permeate throughout the profession.

The editor of the *Dental Cosmos* fails to perceive the relevancy of the quotation given by him, that "the battle can be, *must be*, fought within our own lines." That it is "unintelligible" to him is only another proof that he, in common with many others, is unable to recognize the drift of sentiment, or to be awakened from a lethargy sad to contemplate. There is a spirit, we are happy to believe, abroad in the dental profession, that proposes to fight on and battle bravely until it stands upon a sure foundation, and one of which no man need be ashamed. May the editor of the *Dental Cosmos* rise to the light of the coming day!

